# Therapies for type 2 diabetes: lowering HbA1c and associated cardiovascular risk factors

**DOI:** 10.1186/1475-2840-9-45

**Published:** 2010-08-30

**Authors:** L Romayne Kurukulasuriya, James R Sowers

**Affiliations:** 1 Department of Internal Medicine, Division of Endocrinology, Diabetes and Metabolism, D109 Diabetes Center, UMC, One Hospital Drive, Columbia, MO 65212, USA; 2 Harry S. Truman Memorial Veterans' Hospital, 800 Hospital Drive, Columbia, MO 65201, USA

## Abstract

**Objectives:**

To summarize data supporting the effects of antidiabetes agents on glucose control and cardiovascular risk factors in patients with type 2 diabetes.

**Methods:**

Studies reporting on the effects of antidiabetes agents on glycemic control, body weight, lipid levels, and blood pressure parameters are reviewed and summarized for the purpose of selecting optimal therapeutic regimens for patients with type 2 diabetes.

**Results:**

National guidelines recommend the aggressive management of cardiovascular risk factors in patients with type 2 diabetes, including weight loss and achieving lipid and blood pressure treatment goals. All antidiabetes pharmacotherapies lower glucose; however, effects on cardiovascular risk factors vary greatly among agents. While thiazolidinediones, sulfonylureas, and insulin are associated with weight gain, dipeptidyl peptidase-4 inhibitors are considered weight neutral and metformin can be weight neutral or associated with a small weight loss. Glucagon-like peptide-1 receptor agonists and amylinomimetics (e.g. pramlintide) result in weight loss. Additionally, metformin, thiazolidinediones, insulin, and glucagon-like peptide-1 receptor agonists have demonstrated beneficial effects on lipid and blood pressure parameters.

**Conclusion:**

Management of the cardiovascular risk factors experienced by patients with type 2 diabetes requires a multidisciplinary approach with implementation of treatment strategies to achieve not only glycemic goals but to improve and/or correct the underlying cardiovascular risk factors.

## Introduction

Type 2 diabetes is an increasingly prevalent, complex disease associated with a high risk of morbidity and mortality due to cardiovascular disease (CVD). Approximately 23.6 million Americans have diabetes, with up to 95% having type 2 diabetes [1]. Numerous clinical studies have shown a direct relationship between the level of hyperglycemia and CVD morbidity and mortality. CVD in patients with diabetes includes coronary artery disease, peripheral vascular disease, cerebrovascular disease, diabetic cardiomyopathy, and hypertensive cardiomyopathy [2-5]. A prospective, observational study found a significant relationship between various degrees of hyperglycemia and both microvascular and macrovascular end points, including myocardial infarction (MI) and all-cause mortality (p < 0.0001 for all). Additionally, it has been reported that each 1.0% reduction in glycated hemoglobin (HbA1c) is associated with a 14% reduction in the incidence of MI (p < 0.0001) [6].

However, conflicting findings in large-scale clinical trials involving more than 21,000 patients on the effects of intensive glucose control with aggressive HbA1c goals on CVD events has been a topic of much debate [7]. Two recently published meta analyses of randomized clinical trials reported that intensive glucose control is associated with reductions in CVD events but has no significant effect on CVD death or all-cause mortality. Intensive glucose lowering was associated with a 16-17% reduction in nonfatal MI and an 11-15% reduction in coronary heart disease (CHD) (p < 0.05, for all) [8,9].

Diabetes is a powerful risk factor for the development of atherosclerosis and associated CVD. Patients with diabetes have a 2- to 4-fold higher risk of CVD death compared with patients without diabetes; approximately 65% of deaths in patients with diabetes, of which type 2 diabetes predominates, are a result of CVD [3]. CVD is more likely to develop at a younger age in patients with diabetes than patients without diabetes with increasing risk over the duration of the disease [3]. While pre-menopausal women without diabetes traditionally lag behind men in CVD by approximately 10 years due to the protective effects of estrogen, this benefit is lost by the development of diabetes. The risk of MI in a middle-aged patient with diabetes and no prior MI is the same as that of a patient with a prior MI but no diabetes. Therefore, patients with diabetes are considered to be at high risk for CHD events, equivalent to the risk seen in patients with documented vascular disease [3,10].

The risk of developing CVD is elevated even in those with prediabetes and/or the cardiometabolic syndrome that exhibit impaired fasting glucose (IFG) and/or impaired glucose tolerance (IGT). The approximate annualized risk of nonfatal CV events per 100 patients with IGT or IFG has been estimated at 11.6 to 12.4 and 0.63 to 9.70, respectively [11,12]. Furthermore, the risk of developing CVD dramatically increases when both diabetes and overweight/obesity are present. The coexistence of these 2 comorbidities results in a lifetime risk of between 80% and 90% of development of CVD in women and men, respectively [13]. Nearly 85% of patients with type 2 diabetes are overweight (body mass index [BMI] ≥ 25 kg/m^2^) and approximately 55% are considered obese (BMI ≥ 30 kg/m^2^) [14]. Weight gain and obesity are vital factors in the increasing prevalence of both type 2 diabetes and CVD [14-16]. An analysis of overweight/obese patients with type 2 diabetes (N = 4,916) and baseline BMI 25-40 kg/m^2 ^showed a 13% increased risk of fatal and nonfatal CHD for every 1-unit increase in BMI over a mean 5.6-year follow-up [17].

Implementation of strategies to prevent the development of overt CVD could result in the reduction of a large number of clinical CVD events. Using the Archimedes Model, 11 prevention activities relating to CVD were evaluated to determine their impact on morbidity and mortality. The model was applicable to 156 million Americans, between 20 and 80 years of age, who met the criteria for implementation of CVD prevention strategies, such as weight loss, blood pressure (BP) control, and management of dyslipidemia. If everyone adopted the prevention strategies as outlined, approximately 221 million life-years and 244 million quality-adjusted life-years could be added to adults in the United States over the next 30 years [18]. Due to the strong relationship between type 2 diabetes and CVD, as well as the related morbidity and mortality, optimizing the care of patients with type 2 diabetes and CVD must be a shared responsibility among cardiologists, diabetologists, and primary care physicians, and should include screening for CV risk factors. Throughout the management of patients with type 2 diabetes, strategies should be implemented to improve the CV risk profile [19], including glucose control, which leads to a delay or prevention of vascular complications [6], weight loss, smoking cessation, and management of hypertension and dyslipidemia. Nevertheless, at this time, no specific antiglycemic treatment modality has been shown to lower the incidence of CVD in long-term outcomes trials.

This paper will examine the relationship between type 2 diabetes and CVD, with an emphasis on the effects of antidiabetes agents on glucose lowering and other CVD-associated risk factors, including weight, lipids, BP, prothrombotic factors (e.g fibrinogen) and inflammatory markers (e.g. high-sensitivity C-reactive protein [hs-CRP]).

### Risk Factors, Lifestyle Interventions, and Treatment Guidelines

The non-modifiable and modifiable risk factors for type 2 diabetes and CVD are presented in table 1[10,20]. A joint scientific statement of the American Diabetes Association (ADA) and European Association for the Study of Diabetes (EASD) advocates lifestyle management, including diet and exercise, as the initial treatment approach for the prevention and/or management of type 2 diabetes morbidity and mortality, as well as throughout all stages of type 2 diabetes management, with the goal of weight loss and improvement of modifiable risk factors [21]. Specific dietary recommendations include limiting saturated and trans fat and alcohol intake, monitoring carbohydrate consumption, and increasing dietary fiber. In addition, patients with type 2 diabetes should perform 150 minutes of moderate to vigorous aerobic exercise each week and, in the absence of contraindications, engage in resistance training 3 times a week [20,22].

**Table 1:** Modifiable and non-modifiable risk factors associated with type 2 diabetes mellitus and cardiovascular disease [10,20]

Modifiable Risk Factors	Non-modifiable Risk Factors
Overweight/obesity	Family history of diabetes or premature CHD

Sedentary lifestyle	Cardiovascular disease
Cigarette smoking	Latino/Hispanic, Non-Hispanic black, Asian American, Native American, or Pacific Islander ethnicity

Hypertension	History of gestational diabetes

Increased LDL-C and/or triglycerides and/or low HDL-C	History of delivery of infant with birth weight >9 pounds

Psychiatric illness	Polycystic ovary syndrome

IGT or IFG	Age

CHD, coronary heart disease; HDL-C, high-density lipoprotein cholesterol; IFG, impaired fasting glucose; IGT, impaired glucose tolerance; LDL-C, low-density lipoprotein cholesterol.

Even small weight reductions have been shown to have significant beneficial effects. A weight loss of 5-10% of initial body weight has lowered the risk for diabetes and CVD, as well as significant (p = 0.001) improvements in related modifiable risk factors, including HbA1c, high-density lipoprotein cholesterol (HDL-C), triglycerides (TGs), systolic blood pressure (SBP), and diastolic blood pressure (DBP) [23]. In a meta-analysis of 9 studies including 162 obese patients with type 2 diabetes, a modest 9.6% reduction in initial body weight over 6 weeks was associated with a decrease in fasting plasma glucose concentrations to < 50% of initial values [16].

**Figure 1:**
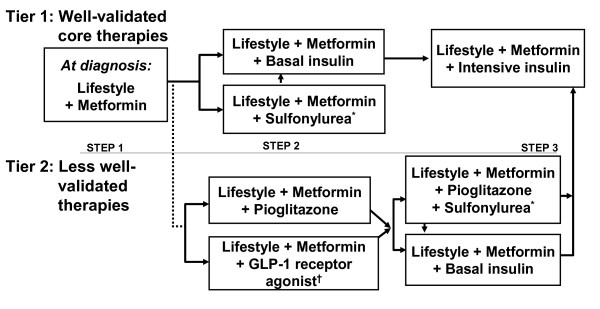
**American Diabetes Association/European Association for the Study of Diabetes consensus guidelines treatment algorithm for patients with type 2 diabetes mellitus**. *Sulfonylureas other than glyburide or chlorpropamide. ^† ^Insufficient clinical use to be confident regarding safety. GLP-1, glucagon like peptide-1. Reprinted with permission from Nathan et al (2009) [21].

Both the American Association of Clinical Endocrinologists (AACE)/American College of Endocrinology (ACE) and ADA have established practice guidelines (with slight differences in goals) outlining target glucose, HbA1c, BP, and lipid concentrations (table 2) [20,22]. Additionally, treatment algorithms have been developed by the ADA/(EASD) and AACE/ACE [24] for the management of patients with type 2 diabetes (figure 1) [21], outlining step-wise recommendations for treatment initiation and alterations based on HbA1c-lowering efficacy, additive effects, and costs associated with interventions [22]. The new AACE/ACE algorithms also prioritize regimens based on their ability to minimize the risk and severity of hypoglycemia and the risk and magnitude of weight gain. Thus, these algorithms favor the use of glucagon-like peptide-1 (GLP-1) receptor agonists and dipeptidyl peptidase-4 (DPP-4) inhibitors because of their efficacy and safety, include thiazolidinediones (TZDs) as "well-validated" agents, and move sulfonylureas (SFUs) to lower priority because of their risk of hypoglycemia, weight gain, and short period of efficacy [24]. At all steps, lifestyle interventions are recommended and should be encouraged [21]. The effects of specific agents on modifiable risk factors and the CV risk profile should also be considered when building an antidiabetes regimen.

**Table 2:** Comparison of guidelines for the management of patients with type 2 diabetes mellitus [20,22]

	HbA1c	Fasting Glucose	Postprandial Glucose	Blood Pressure	Lipids
AACE/ACE, 2007 [20]	≤ 6.5%	Fasting plasma glucose < 110 mg/dL	2-hr postprandial glucose < 140 mg/dL	< 130/80 mmHg	LDL-C < 100 mg/dL (< 70 mg/dL for patients with DM and coronary artery disease) HDL-C >40 mg/dL in men, > 50 mg/dL in women Triglycerides < 150 mg/dL

ADA, 2009 [22]	< 7.0%	Preprandial capillary plasma glucose, 70-130 mg/dL	Peak postprandial capillary plasma glucose < 180 mg/dL	< 130/80 mmHg	LDL-C < 100 mg/dL* HDL-C >40 mg/dL in men, > 50 mg/dL in women Triglycerides < 150 mg/dL

*In individuals with overt cardiovascular disease, a lower LDL-C goal of < 70 mg/dL (1.8 mmol/L), using high doses of a statin, is an option.

AACE, American Association of Clinical Endocrinologists; ACE, American College of Endocrinology; ADA, American Diabetes Association; DM, diabetes mellitus; HbA1c, glycated hemoglobin; HDL-C, high-density lipoprotein cholesterol; LDL-C, low-density lipoprotein cholesterol.

Following are a review of the effects of the various antidiabetic agents on glycemic control, body weight, and other CV risk factors, e.g. serum lipids, BP, prothrombotic factors, and inflammatory markers. While glycemic control is important in reducing a patient's overall CV risk, lipid treatment with statins, management of hypertension, and weight loss have been shown to be more important than just glucose control in reducing CV risk in patients with type 2 diabetes.

### The Effects of Antidiabetes Agents on Glycemic Control

In reviewing the effects of antidiabetic agents on glycemic control, it must be noted that clinical studies may have differences in design, as well as baseline patient characteristics, which may affect results. For example, lower baseline glycemia may reduce the apparent glucose-lowering efficacy of antidiabetic agents. Thus, comparisons of newer agents to older ones is particularly difficult as patients entering more recent clinical trials typically have lower HbA1c than previous studies and do not usually go through a "washout period" prior to drug initiation [25,26]. For the purpose of this paper, individual end points will be analyzed separately and focus more on efficacy than safety parameters.

Data from large-scale clinical trials, including the United Kingdom Prospective Diabetes Study (UKPDS 33, 75, and 80) have shown that reducing hyperglycemia improves morbidity and mortality in patients with type 2 diabetes. In UKPDS 33, patients randomized to intensive therapy (n = 2,729) had lower HbA1c concentrations compared with patients treated with conventional therapy (n = 1,138) over 10 years of follow-up and experienced a lower rate of diabetes-related end points, including a significant 25% reduction in microvascular complications (p = 0.0099) [27]. Likewise, UKPDS 75 found that each 1.0% decrement in HbA1c was associated with a 21% risk reduction in any diabetes-related end point, a 22% reduction in diabetes-related death, and a 14% reduction in all-cause mortality (p < 0.0001 for all) [28]. Furthermore, although differences between HbA1c concentrations were lost after 1 year, the effects of intensive therapy (n = 2,729) versus conventional therapy (n = 1,138) in patients with type 2 diabetes in UKPDS 80 translated into a significant reduction in any diabetes-related end point (9%, p = 0.04), microvascular disease (24%, p = 0.001), diabetes-related death (17%, p = 0.01), and all-cause mortality (13%, p = 0.007) at 10 years [29].

While all currently available antidiabetes agents lower glucose and HbA1c, the magnitude of reduction is variable (table 3) [20,21,25,30-35].

**Table 3:** Therapeutic considerations of selected US FDA-approved antidiabetes agents [20,21,25,30-35]

Intervention	HbA1c Reduction (%)	Effect on Weight	Effect on Lipids	Effect on Blood Pressure	Safety
**Oral**					

SFUs	0.9-2.5	Increased	Small improvements; mainly in TG	Poorly quantified	Increased risk of hypoglycemia

Metformin	1.1-3.0	Neutral or slightly decreased	Improved	Neutral	Contraindicated in patients with renal insufficiency

Glinides	0.4-0.6	Neutral (poorly quantified)	Poorly quantified	Poorly quantified	Caution in patients with hepatic or renal impairment (nateglinide)

TZDs	1.5-1.6	Increased	Improved HDL and TG	Small improvements	Fluid retention, CHF, bone fractures, potential increase in MI (rosiglitazone)

DPP-4 inhibitors	0.8	Neutral	Poorly quantified	Small improvements in non-diabetics	Long-term safety not established

α-Glucosidase inhibitors	0.5-1.0	Suggested decrease	Poorly quantified	Poorly quantified	Frequent flatulence

**Parenteral**					

Insulin	Up to 4.9	Increased	Improved	Neutral	Increased risk of hypoglycemia

GLP-1 receptor agonists	0.8-1.5	Decreased	Improved	Lowered	Nausea and vomiting; hypoglycemia with sulfonylureas; rare pancreatitis and renal dysfunction; thyroid C-cell tumors in rodents

Amylin analog	0.4-0.6	Slightly decreased	Small improvements	Small improvements	Contraindicated in patients with gastroparesis

BID, twice daily; CHF, congestive heart failure; DPP-4, dipeptidyl peptidase-4; GLP-1, glucagon like peptide-1; HbA1c, glycated hemoglobin; HDL, high-density lipoprotein; MI, myocardial infarction; SFUs, sulfonylureas; TG, triglyceride; TZDs, thiazolidinediones; US FDA, United States Food and Drug Administration.

#### Sulfonylureas (SFU)

SFUs lower glucose levels and HbA1c by increasing the secretion of insulin from pancreatic beta-cells, resulting in reduced HbA1c by approximately -0.9% to -2.5%. SFUs are approved for use as monotherapy, as well as in combination with other antidiabetes agents, with the exception of glinides, and insulin. Optimal therapeutic benefits with sulfonylureas are seen at approximately half the maximum dose [20-22].

#### Glinides

Similar to SFUs, glinides stimulate the secretion of insulin; however, because glinides have a shorter half-life, they result in a rapid, short-lived release of insulin for up to 2 hours, thus requiring frequent dosing. The 2 currently available glinides, repaglinide and nateglinide, reduce HbA1c up to -1.9% from baseline and may be used as monotherapy or in combination [20,21,33].

#### Biguanides

Metformin (MET) lowers hyperglycemia by reducing hepatic gluconeogenesis in the presence of insulin and improving insulin sensitivity by increasing peripheral glucose uptake and utilization [20,21,36]. As monotherapy, MET has been associated with reductions in A1C of -1.1% to -3.0% [20]. MET is also approved for use in combination with SFUs, TZDs, insulin [20], and sitagliptin [21].

#### Thiazolidinediones

TZDs affect glucose metabolism in both the liver and periphery through multiple pathways. TZDs are insulin sensitizers and lower glucose by improving the response of target cells to insulin. They also promote adipocyte differentiation, which results in more cells with higher insulin sensitivity, and the expression and translocation of the glucose transporter, GLUT-4, which increases glucose uptake in the presence of insulin [21,37]. The 2 available TZDs, pioglitazone and rosiglitazone, are approved for use in combination with MET, SFUs, glinides, and insulin. These drugs have been shown to produce similar reductions in HbA1c of approximately -1.6%, with reductions ranging from -1.2% to -2.3% over 3-12 months of therapy [20,21,38-41].

#### Insulin

Insulin regulates the metabolism of carbohydrates, protein, and fat by acting on specific membrane-bound receptors on target tissues and facilitating glucose uptake into muscle, adipose, and other tissues [20,36]. Insulin therapy provides the greatest glucose-lowering effects and has been associated with reductions in HbA1c of up to -4.9% when used in combination with MET [21,34]. However, the initiation of insulin therapy is often delayed until the later stages of disease after initial treatment failure due to concerns regarding weight gain, hypoglycemia, and convenience [34].

#### Glucagon-like peptide-1 receptor agonists

GLP-1 receptor agonists are part of a new class of agents, the incretin mimetics, which provide reductions in HbA1c ranging from -0.8% to -1.5%. GLP-1 receptor agonists have many of the effects seen with native GLP-1 in the regulation of glucose metabolism including stimulating insulin production and response during periods of elevated blood glucose, inhibiting glucagon release, slowing nutrient absorption, and increasing feelings of satiety [20]. Exenatide is the first US Food and Drug Administration (FDA)-approved agent in this class for the treatment of patients with type 2 diabetes. It is approved as monotherapy as an adjunct to diet and exercise to improve glycemic control in adults with type 2 diabetes, and is also indicated in combination with SFU, MET, SFU plus MET, or TZD therapy with or without MET [20]. Liraglutide, a once-daily GLP-1 receptor agonist was recently approved by the US FDA. It is indicated as an adjunct to diet and exercise to improve glycemic control in patients with type 2 diabetes. It is not recommended as first-line therapy. A once-weekly formulation of exenatide has been submitted to the US FDA for regulatory review. Exenatide lowers glucose concentrations and HbA1c by approximately -1.0% with reductions of -0.8% to -0.9% at 30 weeks and -1.0% at 3 years (p ≤ 0.001 versus baseline for all) [20,42-47]. The once weekly exenatide formulation has resulted in HbA1c reductions in the range of -1.9% after 30 weeks of therapy [48]. Liraglutide has also shown positive effects on glucose control and reductions in HbA1c ranging from -0.6% to -1.5% over 14 to 52 weeks of therapy, respectively [43,49-52].

#### Dipeptidyl peptidase-4 inhibitors

DPP-4 inhibitors prolong the therapeutic activity of GLP-1 by slowing its metabolism, leading to stimulation of glucose-dependent insulin secretion and inhibition of glucagon secretion. Sitagliptin, the first US FDA-approved DPP-4 inhibitor, has been shown to release up to 80% of DDP-4 activity after oral administration [20,53,54]. The two DDP-4 inhibitors currently available for oral administration are sitagliptin and saxagliptin. Both agents are effective as monotherapy or in combination with other classes and reduce HbA1c by approximately -0.8% [20,55-57].

#### Amylin agonists

Pramlintide is an amylin analog that mimics the action of native amylin, a hormone co-secreted with insulin, and regulates glucose influx through the suppression of glucagon and slowing of gastric emptying. Pramlintide is an injectable agent used in patients who fail to achieve treatment goals on prandial insulin. Treatment with pramlintide has been shown to reduce HbA1c by up to -0.6%, as well as prandial insulin requirements [20].

#### α-Glucosidase inhibitors

α-Glucosidase inhibitors suppress glucose levels by reducing the absorption of carbohydrates from the gastrointestinal tract. They are approved as monotherapy or in combination with SFUs and reduce HbA1c by -0.6% to -1.3% [20].

### The Effects of Antidiabetes Agents on Weight

Many of the available oral antidiabetes agents, as well as insulin, are associated with weight gain that contribute to an increase in CV risk and insulin resistance (table 3) [20,25] thereby creating a significant challenge in the management of overweight/obese patients with type 2 diabetes.

#### Sulfonylureas/insulin

In UKPDS 33, intensive therapy with either SFUs or insulin was associated with significant weight gain compared with patients managed with diet alone (+3.1 kg, p < 0.0001). Patients with type 2 diabetes treated with insulin experienced the greatest increases in body weight (+4.0 kg [p < 0.0001]), followed by chlorpropamide (+2.6 kg [p < 0.001]), and glibenclamide (+1.7 kg [p < 0.001]) when compared with conventional therapy [27].

#### Thiazolidinediones

TZDs have also been associated with weight gain and edema, which has been shown to increase the risk for heart failure. The weight gain associated with TZD therapy is dose-dependent and more dramatic when used in combination with insulin [20,58]. In more than 1,800 patients studied over 16 to 26 weeks, pioglitazone monotherapy was associated with increases of +0.9 to +2.6 kg as doses were uptitrated from 15-45 mg and increases of +2.3 to +4.1 kg in combination with insulin across the same dosing range in patients with type 2 diabetes [58,59]. At doses of 4 and 8 mg, rosiglitazone increases body weight by +1.0 to +3.1 kg when administered as monotherapy and by +4.1 to +5.4 kg when administered with insulin over 6-12 months [58,60].

#### Biguanides

MET therapy is weight neutral in patients with type 2 diabetes and may limit the weight gain experienced with SFU, TZD, or insulin therapy [20,61]. Treatment with MET therapy reported no weight gain when combined with SFUs [62] and slight nonsignificant weight loss when administered as monotherapy (change in BMI, -0.7 kg/m^2 ^over 1 year of therapy) [41]. In 1 study involving 66 patients, MET, in combination with insulin, led to a weight increase of +1.4 kg over 6 months compared with increases of +3.6, +4.6, and +2.9 kg when insulin was administered as monotherapy, in combination with rosiglitazone, or with acarbose, respectively [61].

#### Dipeptidyl peptidase-4 inhibitors

The DDP-4 inhibitors are generally considered weight neutral. Sitagliptin and saxagliptin have been associated with weight reductions ranging from -0.1 to -1.2 kg over 24-30 weeks of monotherapy [55,57,63-65].

#### Glucagon like peptide-1 receptor agonists

The GLP-1 receptor agonists have been associated with weight loss in patients with type 2 diabetes [25]. Exenatide results in progressive, dose-dependant weight loss in patients with type 2 diabetes ranging from -2.8 to -5.3 kg from baseline without a plateau in effect over 3 years of therapy (n = 217, p < 0.0001 at 3 years) [25,31,44,46,66,67], with smaller reductions of -0.8 kg in as early as 2 weeks [68]. At 3 years of follow-up, patients with a baseline BMI < 30 kg/m^2 ^(n = 63) had a reduction of body weight with exenatide of -3.9 kg from baseline (p < 0.0001), and in patients with baseline BMI ≥ 30 kg/m^2 ^(n = 154), exenatide resulted in a weight change of -5.8 kg (p < 0.0001) [45]. Exenatide once weekly results in weight loss of approximately -4.0 kg after 30 weeks, and 75% of patients lost weight [49]. Liraglutide resulted in dose-dependent decreases in body weight ranging from -1.0 to -3.2 kg in more than 1,300 patients with type 2 diabetes treated over 26 weeks [43,51,52].

#### Amylin agonists

Treatment with the amylin analog pramlintide is associated with improved weight control in patients with type 2 diabetes when administered with insulin therapy [20,32,69]. In a *post hoc *analysis of 2 studies in patients with type 2 diabetes, pramlintide (n = 254) or placebo (n = 244) was added to insulin therapy. Therapy with pramlintide resulted in progressive reductions in body weight starting at 2 weeks and a placebo-corrected weight loss of -1.8 kg at 26 weeks (p < 0.0001). Weight loss was most pronounced in patients with a baseline BMI >40 kg/m^2 ^(-3.2 kg) [70].

#### Glinides and α-glucosidase inhibitors

The effects of glinides and α-glucosidase inhibitors on weight have been poorly quantified in patients with type 2 diabetes. In a study of 248 patients (124 patients treated with MET/nateglinide and 124 with MET/glibenclamide) results suggest that the glinide class is weight neutral with no change in BMI occurring over 12 months of therapy in patients treated with nateglinide (+0.4 kg/m^2^) or glibenclamide (+0.4 kg/m^2^) [30]. A Cochrane review of 681 patients summarized the effects of the α-glucosidase inhibitor acarbose on weight, reporting a -1.2 kg weight loss in patients with IFG and/or IGT compared with placebo therapy [35].

### The Effects of Antidiabetes Agents on Other CVD-associated Risk Factors

Patients with type 2 diabetes commonly have decreased HDL-C, increased TGs, normal or slightly elevated low-density lipoprotein cholesterol (LDL-C), and hypertension, all of which contribute to the increased CV risk seen in this patient population. Current guidelines for type 2 diabetes and CVD prevention recognize the importance of aggressively managing these risk factors to minimize the risk of their complications [71-73]. Furthermore, the National Cholesterol Education Program (NCEP) Expert Panel on Detection, Evaluation, and Treatment of High Blood Cholesterol in Adults (Adult Treatment Panel III) consider patients with type 2 diabetes as a CHD risk equivalent to patients with documented CHD and recommend achieving the most stringent LDL-C goal of < 100 mg/dL and consideration for a goal of < 70 mg/dL in very high-risk patients [10,74]. Most of the available antidiabetes agents decrease hyperglycemia but are not associated with significant improvement in BP and dyslipidemia in patients with type 2 diabetes (table 3). Therefore, multiple medications to address both dyslipidemia and hypertension may be required. However, BP control may be a challenge in patients with type 2 diabetes and often requires combination therapy to achieve goals [72].

#### Sulfonylureas

SFUs have been shown to reduce fasting and postprandial TGs, but their effects on other lipid parameters, particularly HDL-C, are inconsistent with some studies showing an increase and others demonstrating a neutral effect [36]. Glimepiride showed minimal changes to lipid parameters during a 12-month study in treatment-naïve patients with type 2 diabetes (total cholesterol [TC], -0.19 mmol/L; HDL-C, -0.01 mmol/L, TG, -0.05 mmol/L) [41]. Likewise, the effects on BP have been poorly quantified, with data coming from several small, poorly controlled trials (total sulfonylurea group < 150 patients) and results ranging from neutral effects to significant increases in BP during treatment with glyburide or glipizide [72]. Over 12 months, glimepiride was associated with small, nonsignificant decreases in SBP (-4.1 mm Hg) and DBP (-4.8 mm Hg) [41]. SFUs generally are not considered to have anti-inflammatory and anti-atherogenic properties. However, gliclazide has demonstrated antioxidant activity which may lead to a small improvement in coagulation, fibrinolysis, and monocyte adhesion to endothelial cells [75,76]. Gliclazide has also been shown to reduce levels of serum intercellular adhesion molecules-1 (ICAM-1) [75]. In a small trial, glimepiride use has resulted in a significant reduction in the levels of tumor necrosis factor-α (TNF-α), interleukin-6 (IL-6), and hs-CRP [77]. Additional studies are needed to define the clinical significance of these findings.

#### Glinides

Clinical trials of patients with type 2 diabetes treated with glinides have shown no significant change in lipids or BP parameters [36]. A recently published, randomized, double-blind study of nateglinide and glibenclamide, in combination with MET, confirmed previous findings [30]. In 119 patients treated with nateglinide over 12 months, there were no significant changes in LDL-C, HDL-C, TGs, SBP, or DBP compared with baseline or SFU therapy at any time point during follow-up [30]. Repaglinide has been reported to have a favorable effect on markers of CV inflammation. IL-6, hs-CRP, and serum ICAM-1 levels have been reported to be reduced in association with repaglinide therapy [78-80]. In addition, endothelial function has been improved [78]. Finally, a randomized trial compared repaglinide with glyburide on carotid intima-media thickness (IMT) after 12 months of therapy. Repaglinide-treated subjects had significantly more frequent regression of IMT than those treated with glyburide (p < 0.01) [81].

#### Biguanides

MET is associated with improvements in lipoprotein metabolism, including decreases in LDL-C, fasting and postprandial TGs, and free fatty acids [20,36]. A meta-analysis of randomized, controlled, clinical trials found that MET lowered BP (SBP and DBP), TGs, TC, and LDL-C, and increased HDL-C from baseline. However, many of these changes did not remain significant when compared with control groups. Data from 37 trials (N = 2,891) were analyzed on the effects of MET on TGs. MET therapy was associated with a reduction of -0.19 mmol/L in TGs, which was significant when compared with the control groups (p = 0.003). Effects on TGs were not affected by baseline BMI [82]. The meta-analysis also found that MET was associated with a nonsignificant increase in HDL-C of +0.01 mmol/L [82], but a significant reduction in LDL-C was reported when compared with controls (-0.22 mmol/L, p < 0.00001) [82]. Data on the effects of MET on BP are variable with studies reporting neutral effects or small decreases in SBP and DBP [72]. Meta-analyses of randomized, controlled clinical trials included 21 trials (n = 1,667) reporting on SBP and 19 trials (n = 1,609) reporting on DBP. Overall, SBP was reduced -1.78 mmHg and DBP -0.57 mmHg; neither reduction was significantly different from the control therapy, even at the highest MET doses [82].

One report with MET has shown a significant effect on a marker of fibrinolysis. In 27 patients treated with MET, titrated up to a dosage of 850 mg three times a day over 12 weeks, plasminogen activator inhibitor-1 (PAI-1) activity significantly fell compared with placebo (p = 0.001) [83]. In a trial dealing with inflammatory markers, patients treated with MET and a TZD (pioglitazone or rosiglitazone) for 12 months had significant reductions of hs-CRP (p < 0.05) in association with reduced insulin resistance [84]. However, a recent study [85] evaluated the effects of initiating insulin glargine or MET or placebo on inflammatory markers in 500 subjects with recently diagnosed (median time from diagnosis 2 years) type 2 diabetes. Levels of hs-CRP, TNF receptor 2, and IL-6 were measured at baseline and after 14 weeks of therapy. Neither insulin nor MET reduced inflammatory marker levels as compared with the placebo groups, and there was no consistent association found between glucose reduction and improvement of inflammatory status [85]. Finally, in 353 subjects with type 2 diabetes, MET or placebo were added to ongoing insulin therapy and their effects on endothelial function and inflammatory markers were evaluated [86]. After 16 weeks of follow-up, MET had no effect on hs-CRP or ICAM-1 levels. However, MET did improve several markers of endothelial function including von Willebrand factor, soluble vascular cell adhesion molecule-1, and soluble E-selectin [86].

#### Thiazolidinediones

While differences may exist among the agents, TZDs are associated with increases in HDL-C, TG reductions, and a modest BP-lowering effect [20,36,72]. In an observational study of 1,170 patients with type 2 diabetes, pioglitazone was associated with significant changes in LDL-C (3.3 versus 3.2 mmol/L, p < 0.001), TGs (2.4 versus 2.2 mmol/L, p = 0.0032), HDL-C (1.3 versus 1.4 mmol/L, p < 0.001), SBP (141.1 versus 137.0 mmHg, p < 0.001), and DBP (82.7 versus 80.0 mmHg, p < 0.001) from baseline after 20 weeks of therapy [87]. A study of 56 patients with type 2 diabetes treated with rosiglitazone (n = 35) or pioglitazone (n = 21) found that both rosiglitazone and pioglitazone significantly increased HDL-C levels (+4% and +2%, respectively, p < 0.01); however, rosiglitazone-associated increases (14%) were significantly higher than those seen in the pioglitazone group (9%, p < 0.05). Additionally, pioglitazone therapy resulted in significantly greater reductions in TGs (-29% versus -5%, p < 0.01) while rosiglitazone was associated with significant increases in LDL-C (+20% versus +1%, p < 0.05) [39]. In a small study of 22 nondiabetic subjects, pioglitazone significantly reduced DBP (83 versus 77 mmHg, p = 0.02) and TGs (1.5 versus 1.0, p = 0.02) while rosiglitazone increased TC (4.7 versus 5.1 mmol/L, p = 0.047) and LDL-C levels (2.7 versus 3.1 mmol/L, p = 0.07) [88].

TZDs have shown beneficial effects on a number of markers of CV risk. In the Insulin Resistance Intervention after Stroke (IRIS) V study, the effects of pioglitazone over 20 weeks on levels of hs-CRP were studied in 1,170 subjects naïve to TZD therapy. A decrease of hs-CRP levels from a mean baseline of 3.8 μM to 2.8 μM was significant (p < 0.01) and occurred in association with improvement of HbA1c, BP, and serum lipids [87]. Miyazaki and DeFronzo (2008) studied rosiglitazone (n = 35) and pioglitazone (n = 21) and their effects on adipocytokines (TNF-α, leptin, and adiponectin) in patients with type 2 diabetes treated for 12 weeks. Both drugs significantly decreased TNF-α (p < 0.05) and increased adiponectin levels (p < 0.01). Leptin levels did not change with either agent [39]. These beneficial changes were in association with a reduction of insulin resistance and improved glycemic control.

The Pioneer study consisted of 192 subjects with type 2 diabetes who were enrolled in an open-label, 6-month study of pioglitazone or glimepiride to assess the anti-inflammatory and antiatherogenic effects of each agent. Pioglitazone, but not glimepiride, significantly improved hs-CRP levels (p < 0.05, as well as levels of monocyte chemo-attractant protein-1 and matrix metalloproteinases-9 (p < 0.05 for both). Also, pioglitazone caused substantial regression of carotid IMT from baseline (p < 0.001) [89]. Finally, 3 recent articles have described the beneficial effects of the TZDs including reductions of hs-CRP, improved endothelial function, reduced procoagulatory state, and clinical outcome trials showing reduction of restenosis and reocclusion rates post percutaneous coronary intervention [90-92].

#### Insulin

While insulin is effective in decreasing fasting and postprandial TG levels, a systematic review of the effects of insulin on HDL-C levels found variable results with clinical studies reporting increases or a neutral effect [36]. In a study of 104 insulin-naïve patients receiving combination therapy of SFU and MET, the addition of insulin resulted in reductions in TC (-4.4%), LDL-C (-1.4%) and TG (-19.0%) with no change in HDL-C levels [93]. Insulin secretion is thought to increase BP, although there is little scientific evidence that insulin has deleterious effects on BP parameters [72]. Two recent *post hoc *analyses (combined N = 2,065) reported nonsignificant changes in both SBP (-0.3 to -0.5 mmHg) and DBP (-0.5 to -0.9 mmHg) from baseline over approximately 6 months of insulin therapy [94,95].

As noted above, a recent study [85] evaluated the effects of initiating insulin glargine or MET or placebo on inflammatory markers in 500 subjects with recently diagnosed (median time from diagnosis 2 years) type 2 diabetes. Neither insulin nor MET reduced inflammatory marker levels as compared with the placebo groups, and there was no consistent association found between glucose reduction and improvement of inflammatory status [85]. Similarly, the ability of insulin glargine to reduce levels of hs-CRP and PAI-1 was evaluated in 40 subjects with type 2 diabetes inadequately controlled with MET and SFU. After 24 weeks, insulin glargine had no effect on levels of hs-CRP or PAI-1 [96].

#### Glucagon-like peptide-1 receptor agonists

Unlike most oral antidiabetes agents, exenatide has shown beneficial effects on lipid profiles, including TC and LDL [46,66,67,94,95]. After 16 weeks of exenatide therapy in patients with type 2 diabetes, patients with the metabolic syndrome experienced significant reductions in TC (-7.4 mg/dL, p < 0.001), TGs (-16.7 mg/dL, p < 0.001), SBP (-2.6 mmHg, p < 0.01), and DBP (-1.2 mmHg, p < 0.03), as well as insignificant changes in LDL-C (-2.8 mg/dL) and HDL-C (-1.4 mg/dL) [67]. In a *post hoc *analysis of an open-label extension trial, 151 patients treated with exenatide for 3.5 years experienced significant improvements in TGs (-12%, p = 0.0003), TC (-5%, p = 0.0007), HDL-C (+24%, p < 0.0001), SBP (-2%, p = 0.0063), and DBP (-4%, p < 0.0001) [46].

Exenatide was given once weekly to 120 patients who were followed for 52 weeks. SBP decreased -6.2 mmHg and DBP decreased -2.8 mmHg from a baseline of 128/78 mmHg, p < 0.05 for both. In patients with SBP >130 mmHg, reductions of SBP and DBP were even greater (-11.4 mmHg and -3.6 mmHg, respectively, p < 0.05 for both). These BP changes appeared to be independent of weight loss or concomitant BP medication changes. Favorable improvements in serum lipid profiles were also reported. TC decreased -7.9 mg/dL from baseline of 170 mg/dL (CI: -13.7, -2.0), LDL-C decreased -2.2 mg/dL from a baseline of 89 mg/dL (CI: -6.9, 2.5), TG decreased -40 mg/dL from a baseline of 197 mg/dL (CI: -62.8, -17.3), and HDL-C decreased -0.3 mg/dL from a baseline of 44 mg/dL (CI: -1.9, 1.3) [97].

A recent study by Derosa and colleagues (2010) [98] compared the effects of exenatide versus glibenclamide on glycemic control, body weight, beta-cell function, insulin resistance, and inflammatory state in 128 patients with type 2 diabetes. Patients were inadequately controlled with MET and were randomized to exenatide titrated to 10 μg subcutaneous BID or glibenclamide 5 mg three times a day for 12 months. Hs-CRP was significantly improved with exenatide (from a baseline of 1.9 mg/L to 1.5 mg/L) compared with glibenclamide (from a baseline of 1.9 mg/L to 1.8 mg/L, [p < 0.05 versus glibenclamide]). Resistin levels and levels of retinol binding protein-4 were decreased with exenatide and increased with glibenclamide (p < 0.01 versus glibenclamide). These changes were associated with improvements of glycemic control, insulin resistance, and beta-cell function [98].

One study compared liraglutide 1.8 mg once a day (n = 233) with exenatide 10 μg twice a day (n = 231) with a follow-up of 26 weeks (Liraglutide Effect and Action in Diabetes [LEAD-6]). Compared with exenatide, liraglutide significantly reduced TG (-0.41 versus -0.23 mmol/L, p < 0.04) and free fatty acids (-0.17 versus -0.10 mmol/L, p < 0.001). There were no significant differences between the 2 groups on the effects on TC (-0.20 versus -0.09 mmol/L), LDL-C (-0.44 versus -0.40 mmol/L), or HDL-C (-0.04 versus -0.05 mmol/L) [43].

A second study compared liraglutide 1.2 mg per day (n = 178) or 1.8 mg per day (n = 178) to placebo (n = 177) in patients with type 2 diabetes receiving MET and rosiglitazone. Liraglutide 1.2 mg significantly reduced LDL-C (-0.28 versus -0.10 mmol/L, p < 0.05), TG (-0.28 versus -0.13 mmol/L, p < 0.05), and free fatty acids (-0.03 versus +0.02 mmol/L, p < 0.05) as compared with placebo [52]. Liraglutide is also associated with reductions in SBP ranging from -2.1 to -6.7 mmHg and DBP from -1.05 to -2.3 mmHg over 26 to 52 weeks of therapy [43,49,52].

Liraglutide's effects on CV risk markers were evaluated in 165 patients with type 2 diabetes on oral therapy. Subjects were randomized to liraglutide or placebo and followed for 14 weeks. Liraglutide resulted in a significant decrease in PAI-1 levels (p = 0.045) with nonsignificant reductions in hs-CRP. There were no treatment effects on levels of adiponectin, leptin, IL-6, or TNF-α [99].

#### Dipeptidyl peptidase-4 inhibitors

The effects of DDP-4 inhibitors on CV risk factors are well documented. In a systematic review and meta-analysis of incretin therapy in patients with type 2 diabetes, Amori et al (2007) summarized 13 trials reporting data on body weight in patients receiving DDP-4 inhibitors. The data showed that DDP-4 inhibitors produce a small increase in weight when compared with placebo and had a small but favorable effect when compared with SFUs or TZDs [25]. Similarly, 14 trials evaluating the effects of DPP-4 on lipid levels showed no consistent effect but there was an overall favorable trend in levels of TGs and HDL-C and LDL-C [25]. In a study of sitagliptin added to MET therapy, sitagliptin was associated with reductions in TGs (-4.8%, p ≤ 0.05 versus placebo) and increases in HDL-C (+4.3%), LDL-C (+11.4%), and TC (+4.9%, p ≤ 0.05 versus placebo) over 26 weeks of therapy in patients with type 2 diabetes [65]. Raz et al (2008) showed that sitagliptin had no significant between-group differences in fasting blood lipid levels or body weight as compared with placebo in 521 patients with type 2 diabetes treated for 30 weeks [57]. In a study of 19 patients with mild to moderate hypertension on stable antihypertensive medication and without diabetes, sitagliptin produced small reductions in 24-hour SBP (-2.0 to -2.2 mmHg) and DBP (-1.6 to -1.8 mmHg) over 5 days of therapy, which were significantly different from placebo (p < 0.05) [100].

#### Amylin agonists

The effects of amylin analogs on lipid parameters appear to be modest and dose-dependent. In a 4-week study of 203 patients with type 2 diabetes currently receiving insulin therapy, pramlintide was associated with reductions in TC (-4.7 to -10.5 mg/dL), LDL-C (-4.4 to -.75 mg/dL), and TG (-9.0 to -66.4 mg/dL), with the greatest reductions seen when the dosage was increased from 120 to 240 mg/day. Minimal effects on HDL-C were reported (-0.51 to -0.70 mg/dL) [69]. Although the data are limited, clinical trials show that pramlintide is not associated with increases in BP in patients with type 2 diabetes [32].

#### α-Glucosidase Inhibitors

A Cochrane review of the literature involving more than 600 patients in the literature reported small, nonsignificant changes in TC (-0.13 mmol/L), LDL-C (-0.16 mmol/L), TGs (-0.18 mmol/L), and HDL-C (0.09 mmol/L), as well as SBP (0.03 mmHg) and DBP (-1.31 mmHg) [35]. A study in 132 subjects with IGT was randomized to placebo or acarbose with a mean follow-up of 3.9 years. A significant reduction of the progression of carotid IMT was observed in the acarbose arm at the end of follow-up (p = 0.027). The annual progression of IMT was reduced by 50% [101]. In the Study to Prevent Non-Insulin-Dependent Mellitus (STOP-NIDDM), acarbose was found to reduce the relative risk of developing any CV event by 49% (p = 0.03) with an absolute risk reduction of 2.5% [102].

In summary, the SFUs, insulin, and TZDs are generally associated with weight gain. The glinides, and DPP-4 inhibitors are weight neutral, MET is weight neutral or is associated with some weight loss and the GLP-1 receptor agonists are associated with significant weight reductions (the amylin analogs and the α-glucosidase inhibitors less so). Insulin, the TZDs, MET, and the GLP-1 receptor agonists show fairly robust effects on lipid levels in patients with type 2 diabetes. The other agents generally have neutral or insignificant effects. Finally, most antidiabetes agents have shown small improvements or are neutral with regard to effects on BP while the GLP-1 receptor agonists have been shown to have significant effects on SBP and DBP. It should also be noted that while beneficial effects on CVD surrogate end points have been reported, there are no data demonstrating a reduction of the incidence of CVD end points.

## Conclusions

Overweight/obese patients with type 2 diabetes are at increased risk for CVD. The majority of patients with type 2 diabetes are overweight/obese, which contributes to an even higher risk for CVD. Exacerbating this concern is the fact many antidiabetes therapies are associated with weight gain. It is of utmost importance that a multidisciplinary approach be undertaken to aggressively manage modifiable CV risk factors in patients with type 2 diabetes to prevent the associated morbidity and mortality that is highly prevalent.

All antidiabetes pharmacotherapies lower glucose; however, the effects on modifiable CV risk factors, such as lipid and BP parameters, vary greatly among agents. While some therapies may lead to significant weight gain, others may have a weight-neutral effect or result in weight loss. Most antidiabetes agents do not have a significant adverse effect on BP or lipid parameters. GLP-1 receptor agonists lower HbA1c and body weight and have shown beneficial effects on lipid and BP parameters. These factors should be taken into consideration when selecting an individualized antidiabetes regimen.

## Competing interests

Dr. Kurukulasuriya declares: None

Dr Sowers declares: receiving an NIH grant and Harry S Truman VA grant; has served on Advisory Boards for Forest Research Laboratories and Novartis Pharmaceuticals

## Authors' contributions

LRK and JRS were involved in the discussion of the concept of this article, directed the content of the initial outline, revised it critically for important intellectual content, and provided final approval of the manuscript.
